# 2,3-Di­ethyl­benzo[*g*]quinoxaline

**DOI:** 10.1107/S241431462000454X

**Published:** 2020-04-07

**Authors:** Guy Crundwell, Ashley Leeds

**Affiliations:** a Central Connecticut State University, Department of Chemistry & Biochemistry, 1619 Stanley Street, New Britain, CT 06050, USA; b Department of Chemistry & Biochemistry, Central Connecticut State University, 1619 Stanley Street, New Britain, CT 06053, USA; University of Aberdeen, Scotland

**Keywords:** crystal structure, benzoquinoxaline

## Abstract

The title compound, C_16_H_16_N_2_, was synthesized by dispersing 3,4-hexa­nedione in a methanol–water solution containing the acid catalyst NH_4_HF_2_, then adding 1,2-di­aminona­phthalene. The fused-ring system of the title compound is close to planar (r.m.s. deviation = 0.028 Å); one of the pendant methyl C atoms lies close to the ring plane [deviation = 0.071 (2) Å; N—C—C—C = −0.27 (18)°] whereas the other is significantly displaced [–1.7136 (18) Å; 91.64 (16)°]. The mol­ecules pack in space group *I*  in a distinctive criss-cross motif supported by numerous aromatic π–π stacking inter­actions [shortest centroid–centroid separation = 3.5805 (6) Å].

## Abstract 2

In the title compound, one of the pendant methyl groups lies close to the fused-ring plane and the other is significantly displaced.

## Graphical abstract

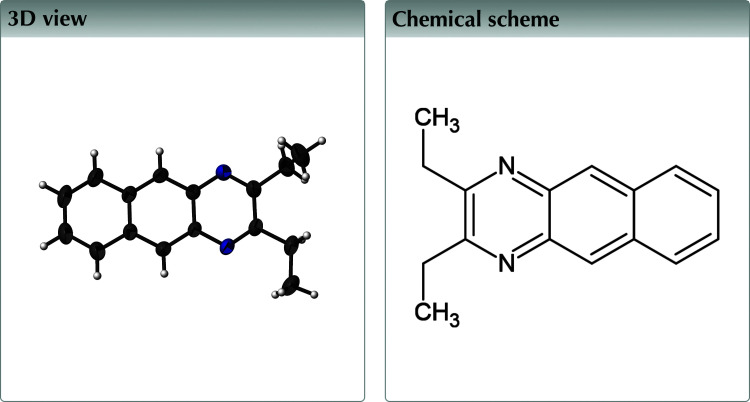

## Structure description

**Figure 1:**
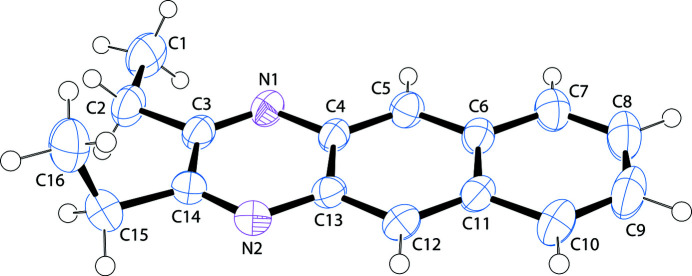
A view of the title compound with displacement ellipsoids drawn at the 50% probability level.

The bond lengths and angles in the title compound fall within their expected values and the C3–C14/N1/N2 fused-ring system is close to planar (r.m.s. deviation = 0.028 Å). The C1 methyl atom lies close to the ring plane [deviation = 0.071 (2) Å; N1—C3—C2—C1 = −0.027 (16)°] whereas C16 is significantly displaced [deviation = −1.7136 (18) Å; N2—C14—C15—C16 = 91.64 (16)°] (Fig. 1 ▸).

**Figure 2:**
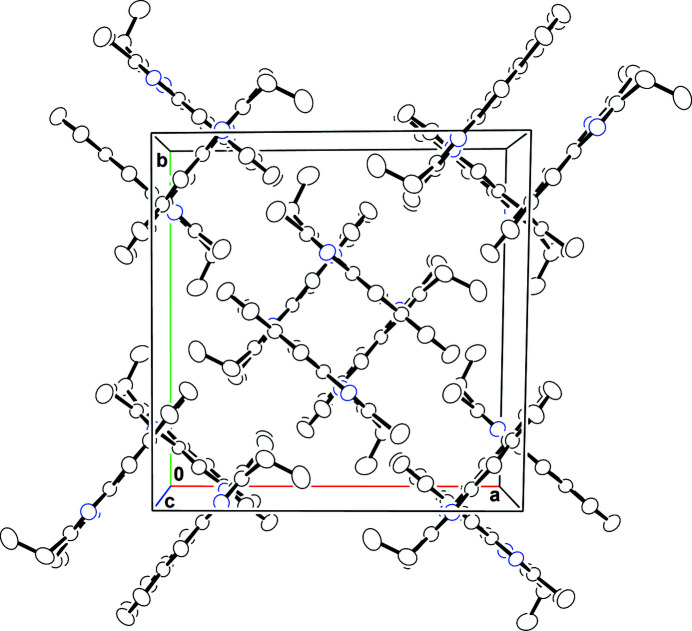
A view of the unit cell of the title compound along [001].

In the crystal, the molecles pack in a distinctive criss-cross motif (Fig. 2 ▸) in space group *I* 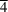 with stacks of mol­ecules propagating in the [001] direction. Numerous aromatic π–π stacking inter­actions help to consolidate the packing [shortest centroid–centroid separation = 3.5805 (6) Å].

## Synthesis and crystallization

2,3-Di­ethyl­benzo[*g*]quinoxaline, C_16_H_16_N_2_, was prepared using the method used by Lassagne *et al.* (2015 ▸) to create 2,3-di­aryl­pyrido­pyrazines. In a 50-ml Erlenmeyer flask equipped with a stir bar, 10.0 mmol of hexa­nedione (1.14 g) was dispersed in 20 ml of a 2.5 × 10^−3^ *M* NH_4_HF_2_ solution in MeOH and 2 ml of distilled water. To that stirred solution, 10.0 mmol of 1,2-naphthalenedi­amine (1.58 g) was added. The solution was allowed to stir overnight despite evidence of product after the first hour: 1.44 grams of a pale whitish powder was filtered and washed with two 2 ml aliquots of ice-cold methanol (60.9% yield). The crude product was mostly pure by NMR but was further purified by recrystallization from a 50:50 methanol/toluene solution (1.11 g recovered, 47.0% yield overall). (m.p. 411 K) ATR–IR (cm^−1^) 2981, 2934, 1703, 1575, 1455, 1351, 1325, 910, 889, 754; ^1^H NMR (300 MHz, CDCl_3_): δ 8.58 (*s*, 1H), 8.07 (*m*, 1H), 7.55 (*m*, 1H) 3.09 (*q*, 2H), 1.49 (*t*, 3H); ^13^C (300 MHz, CDCl_3_): δ 158.14, 138.06, 133.24, 128.35, 126.57, 126.15, 28.63, 12.07. Crystals for the diffraction experiment were grown from slow evaporation of a methyl­ene chloride solution. FTIR, ^1^H NMR, and ^13^C NMR spectra are given as supporting information.

## Refinement

Crystal data, data collection and structure refinement details are summarized in Table 1 ▸. The absolute structure of the crystal chosen for data collection was indeterminate in the refinement reported here.

## Supplementary Material

Crystal structure: contains datablock(s) I. DOI: 10.1107/S241431462000454X/hb4346sup1.cif

Structure factors: contains datablock(s) I. DOI: 10.1107/S241431462000454X/hb4346Isup2.hkl

Supporting information file. DOI: 10.1107/S241431462000454X/hb4346Isup4.cml

Supplementary Material (FTIR, 1H NMR, 13C NMR). DOI: 10.1107/S241431462000454X/hb4346sup3.pdf

CCDC reference: 1994287

Additional supporting information:  crystallographic information; 3D view; checkCIF report

## Figures and Tables

**Table 1:** Experimental details

Crystal data	
Chemical formula	C_16_H_16_N_2_
*M*_r_	236.31
Crystal system, space group	Tetragonal, *I* 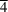
Temperature (K)	293
*a*, *c* (Å)	13.93535 (18), 13.1629 (3)
*V* (Å^3^)	2556.16 (7)
*Z*	8
Radiation type	Mo *K*α
μ (mm^−1^)	0.07
Crystal size (mm)	0.39 × 0.33 × 0.27

Data collection	
Diffractometer	Rigaku Xcalibur, Sapphire3
Absorption correction	Multi-scan (*CrysAlis PRO*; Rigaku, 2018 ▸)
*T*_min_, *T*_max_	0.922, 1.000
No. of measured, independent and observed [*I* > 2σ(*I*)] reflections	31103, 4779, 3869
*R*_int_	0.031
(sin θ/λ)_max_ (Å^−1^)	0.778

Refinement	
*R*[*F* ^2^ > 2σ(*F* ^2^)], *wR*(*F* ^2^), *S*	0.048, 0.136, 1.04
No. of reflections	4779
No. of parameters	165
H-atom treatment	H-atom parameters constrained
Δρ_max_, Δρ_min_ (e Å^−3^)	0.28, −0.14

Computer programs: *CrysAlis PRO* (Rigaku, 2018 ▸), *SHELXM* (Sheldrick, 2008 ▸), *SHELXL* (Sheldrick, 2008 ▸) and *OLEX2* (Dolomanov *et al.*, 2009 ▸).

## full crystallographic data

### Crystal data

**Table:**

C_16_H_16_N_2_	Melting point: 411 K
*M**_r_* = 236.31	Mo *K*α radiation, λ = 0.71073 Å
Tetragonal, *I*4	Cell parameters from 7488 reflections
*a* = 13.93535 (18) Å	θ = 4.6–31.8°
*c* = 13.1629 (3) Å	µ = 0.07 mm^−^^1^
*V* = 2556.16 (7) Å^3^	*T* = 293 K
*Z* = 8	Block, white
*F*(000) = 1008	0.39 × 0.33 × 0.27 mm
*D*_x_ = 1.228 Mg m^−^^3^	

### Data collection

**Table:**

Rigaku Xcalibur, Sapphire3 diffractometer	4779 independent reflections
Radiation source: fine-focus sealed X-ray tube, Enhance (Mo) X-ray Source	3869 reflections with *I* > 2σ(*I*)
Graphite monochromator	*R*_int_ = 0.031
Detector resolution: 16.1790 pixels mm^-1^	θ_max_ = 33.6°, θ_min_ = 4.3°
ω scans	*h* = −21→21
Absorption correction: multi-scan (CrysAlisPro; Rigaku, 2018)	*k* = −21→21
*T*_min_ = 0.922, *T*_max_ = 1.000	*l* = −20→19
31103 measured reflections	

### Refinement

**Table:**

Refinement on *F*^2^	Secondary atom site location: difference Fourier map
Least-squares matrix: full	Hydrogen site location: inferred from neighbouring sites
*R*[*F*^2^ > 2σ(*F*^2^)] = 0.048	H-atom parameters constrained
*wR*(*F*^2^) = 0.136	*w* = 1/[σ^2^(*F*_o_^2^) + (0.0756*P*)^2^ + 0.2732*P*] where *P* = (*F*_o_^2^ + 2*F*_c_^2^)/3
*S* = 1.04	(Δ/σ)_max_ < 0.001
4779 reflections	Δρ_max_ = 0.28 e Å^−^^3^
165 parameters	Δρ_min_ = −0.14 e Å^−^^3^
0 restraints	Absolute structure: Flack H D (1983), Acta Cryst. A39, 876-881
Primary atom site location: dual	Absolute structure parameter: 0 (2)

### Special details

**Table:**

Geometry. All esds (except the esd in the dihedral angle between two l.s. planes) are estimated using the full covariance matrix. The cell esds are taken into account individually in the estimation of esds in distances, angles and torsion angles; correlations between esds in cell parameters are only used when they are defined by crystal symmetry. An approximate (isotropic) treatment of cell esds is used for estimating esds involving l.s. planes.
Refinement. Refinement of F^2^ against ALL reflections. The weighted R-factor wR and goodness of fit S are based on F^2^, conventional R-factors R are based on F, with F set to zero for negative F^2^. The threshold expression of F^2^ > 2sigma(F^2^) is used only for calculating R-factors(gt) etc. and is not relevant to the choice of reflections for refinement. R-factors based on F^2^ are statistically about twice as large as those based on F, and R- factors based on ALL data will be even larger. H atoms were included in calculated positions with C—H = 0.93–0.97 Å and refined as riding atoms with *U*_iso_ = 1.2*U*_eq_(C) or 1.5*U*_eq_(methyl C). Reflections affected by the beam stop were omitted from the refinement.

### Fractional atomic coordinates and isotropic or equivalent isotropic displacement parameters (Å^2^)

**Table:**

	*x*	*y*	*z*	*U*_iso_*/*U*_eq_	
C1	−0.29561 (13)	−1.16936 (12)	−0.44274 (13)	0.0600 (4)	
H1A	−0.3206	−1.1110	−0.4711	0.090*	
H1B	−0.3333	−1.2227	−0.4659	0.090*	
H1C	−0.2302	−1.1776	−0.4640	0.090*	
C2	−0.29974 (10)	−1.16422 (10)	−0.32887 (12)	0.0493 (3)	
H2A	−0.2793	−1.2256	−0.3016	0.059*	
H2B	−0.3660	−1.1546	−0.3087	0.059*	
C3	−0.23964 (8)	−1.08656 (8)	−0.28133 (10)	0.0379 (2)	
C4	−0.13277 (8)	−0.96118 (8)	−0.29463 (9)	0.0328 (2)	
C5	−0.07941 (9)	−0.89899 (9)	−0.35476 (9)	0.0378 (2)	
H5	−0.0815	−0.9047	−0.4251	0.045*	
C6	−0.02254 (8)	−0.82781 (8)	−0.31001 (9)	0.0360 (2)	
C7	0.03189 (10)	−0.76181 (10)	−0.36928 (12)	0.0475 (3)	
H7	0.0310	−0.7661	−0.4398	0.057*	
C8	0.08527 (12)	−0.69247 (11)	−0.32317 (15)	0.0567 (4)	
H8	0.1199	−0.6493	−0.3627	0.068*	
C9	0.08872 (12)	−0.68520 (11)	−0.21668 (15)	0.0608 (4)	
H9	0.1253	−0.6372	−0.1867	0.073*	
C10	0.03930 (10)	−0.74736 (11)	−0.15731 (12)	0.0515 (3)	
H10	0.0430	−0.7421	−0.0870	0.062*	
C11	−0.01854 (8)	−0.82097 (9)	−0.20173 (10)	0.0369 (2)	
C12	−0.07119 (8)	−0.88458 (9)	−0.14191 (9)	0.0380 (2)	
H12	−0.0676	−0.8806	−0.0715	0.046*	
C13	−0.12889 (8)	−0.95376 (8)	−0.18678 (8)	0.0333 (2)	
C14	−0.23818 (8)	−1.07610 (9)	−0.17124 (10)	0.0381 (2)	
C15	−0.30438 (11)	−1.13340 (11)	−0.10459 (12)	0.0522 (3)	
H15A	−0.2763	−1.1398	−0.0375	0.063*	
H15B	−0.3123	−1.1972	−0.1328	0.063*	
C16	−0.40207 (12)	−1.08493 (14)	−0.09594 (16)	0.0684 (5)	
H16A	−0.4299	−1.0786	−0.1623	0.103*	
H16B	−0.3945	−1.0225	−0.0661	0.103*	
H16C	−0.4434	−1.1231	−0.0539	0.103*	
N1	−0.18907 (7)	−1.03067 (7)	−0.34033 (9)	0.0388 (2)	
N2	−0.18387 (7)	−1.01278 (8)	−0.12633 (8)	0.0388 (2)	

### Atomic displacement parameters (Å^2^)

**Table:**

	*U*^11^	*U*^22^	*U*^33^	*U*^12^	*U*^13^	*U*^23^
C1	0.0616 (8)	0.0543 (8)	0.0641 (10)	−0.0115 (7)	−0.0117 (8)	−0.0176 (7)
C2	0.0434 (6)	0.0432 (7)	0.0613 (9)	−0.0115 (5)	−0.0068 (6)	−0.0058 (6)
C3	0.0336 (5)	0.0337 (5)	0.0463 (6)	−0.0024 (4)	−0.0068 (5)	−0.0029 (4)
C4	0.0293 (5)	0.0313 (5)	0.0377 (5)	−0.0020 (4)	−0.0033 (4)	−0.0039 (4)
C5	0.0381 (5)	0.0396 (6)	0.0358 (5)	−0.0027 (4)	−0.0006 (4)	−0.0041 (4)
C6	0.0306 (5)	0.0336 (5)	0.0439 (6)	−0.0011 (4)	0.0009 (4)	−0.0024 (4)
C7	0.0441 (6)	0.0445 (6)	0.0539 (8)	−0.0059 (5)	0.0057 (6)	0.0018 (6)
C8	0.0494 (8)	0.0470 (7)	0.0738 (11)	−0.0151 (6)	0.0073 (7)	0.0010 (7)
C9	0.0510 (8)	0.0494 (8)	0.0820 (12)	−0.0184 (6)	−0.0056 (8)	−0.0122 (8)
C10	0.0467 (7)	0.0515 (7)	0.0563 (8)	−0.0122 (6)	−0.0081 (6)	−0.0113 (6)
C11	0.0302 (5)	0.0360 (5)	0.0446 (6)	0.0000 (4)	−0.0049 (4)	−0.0058 (4)
C12	0.0365 (6)	0.0423 (6)	0.0353 (5)	−0.0022 (4)	−0.0059 (4)	−0.0042 (4)
C13	0.0292 (5)	0.0340 (5)	0.0368 (5)	0.0016 (4)	−0.0041 (4)	0.0006 (4)
C14	0.0341 (5)	0.0356 (5)	0.0445 (6)	0.0005 (4)	−0.0041 (5)	0.0067 (5)
C15	0.0550 (8)	0.0455 (7)	0.0561 (8)	−0.0093 (6)	−0.0022 (6)	0.0152 (6)
C16	0.0504 (8)	0.0754 (11)	0.0794 (11)	−0.0118 (8)	0.0130 (8)	0.0169 (9)
N1	0.0364 (5)	0.0390 (5)	0.0409 (5)	−0.0054 (4)	−0.0047 (4)	−0.0056 (4)
N2	0.0384 (5)	0.0402 (5)	0.0378 (5)	−0.0016 (4)	−0.0044 (4)	0.0044 (4)

### Geometric parameters (Å, º)

**Table:**

C1—H1A	0.9600	C8—H8	0.9300
C1—H1B	0.9600	C8—C9	1.406 (3)
C1—H1C	0.9600	C9—H9	0.9300
C1—C2	1.502 (2)	C9—C10	1.355 (2)
C2—H2A	0.9700	C10—H10	0.9300
C2—H2B	0.9700	C10—C11	1.4296 (16)
C2—C3	1.5047 (17)	C11—C12	1.3943 (17)
C3—C14	1.4566 (18)	C12—H12	0.9300
C3—N1	1.3062 (16)	C12—C13	1.3873 (15)
C4—C5	1.3894 (15)	C13—N2	1.3772 (15)
C4—C13	1.4245 (15)	C14—C15	1.5027 (18)
C4—N1	1.3839 (13)	C14—N2	1.3042 (16)
C5—H5	0.9300	C15—H15A	0.9700
C5—C6	1.3997 (16)	C15—H15B	0.9700
C6—C7	1.4247 (17)	C15—C16	1.524 (2)
C6—C11	1.4296 (17)	C16—H16A	0.9600
C7—H7	0.9300	C16—H16B	0.9600
C7—C8	1.362 (2)	C16—H16C	0.9600
			
H1A—C1—H1B	109.5	C10—C9—C8	120.77 (14)
H1A—C1—H1C	109.5	C10—C9—H9	119.6
H1B—C1—H1C	109.5	C9—C10—H10	119.7
C2—C1—H1A	109.5	C9—C10—C11	120.63 (14)
C2—C1—H1B	109.5	C11—C10—H10	119.7
C2—C1—H1C	109.5	C6—C11—C10	118.53 (12)
C1—C2—H2A	108.4	C12—C11—C6	120.01 (10)
C1—C2—H2B	108.4	C12—C11—C10	121.46 (12)
C1—C2—C3	115.34 (12)	C11—C12—H12	119.8
H2A—C2—H2B	107.5	C13—C12—C11	120.42 (10)
C3—C2—H2A	108.4	C13—C12—H12	119.8
C3—C2—H2B	108.4	C12—C13—C4	119.79 (10)
C14—C3—C2	119.57 (11)	N2—C13—C4	120.75 (10)
N1—C3—C2	118.81 (11)	N2—C13—C12	119.44 (10)
N1—C3—C14	121.62 (10)	C3—C14—C15	121.27 (11)
C5—C4—C13	120.14 (10)	N2—C14—C3	121.79 (11)
N1—C4—C5	119.49 (10)	N2—C14—C15	116.81 (12)
N1—C4—C13	120.37 (10)	C14—C15—H15A	109.5
C4—C5—H5	119.8	C14—C15—H15B	109.5
C4—C5—C6	120.35 (10)	C14—C15—C16	110.89 (12)
C6—C5—H5	119.8	H15A—C15—H15B	108.1
C5—C6—C7	121.91 (11)	C16—C15—H15A	109.5
C5—C6—C11	119.27 (11)	C16—C15—H15B	109.5
C7—C6—C11	118.82 (11)	C15—C16—H16A	109.5
C6—C7—H7	119.8	C15—C16—H16B	109.5
C8—C7—C6	120.31 (13)	C15—C16—H16C	109.5
C8—C7—H7	119.8	H16A—C16—H16B	109.5
C7—C8—H8	119.5	H16A—C16—H16C	109.5
C7—C8—C9	120.93 (14)	H16B—C16—H16C	109.5
C9—C8—H8	119.5	C3—N1—C4	117.69 (10)
C8—C9—H9	119.6	C14—N2—C13	117.71 (10)

## References

[bb1] Dolomanov, O. V., Bourhis, L. J., Gildea, R. J., Howard, J. A. K. & Puschmann, H. (2009). *J. Appl. Cryst.* **42**, 339–341.

[bb3] Lassagne, F., Chevallier, F., Roisnel, T., Dorcet, V., Mongin, F. & Domingo, L. R. (2015). *Synthesis*, **47**, 2680–2689.

[bb4] Rigaku (2018). *CrysAlis PRO*. Rigaku Inc., Tokyo, Japan.

[bb5] Sheldrick, G. M. (2008). *Acta Cryst.* A**64**, 112–122. 10.1107/S0108767307043930 18156677

