# Supplementary material for: 2,3-Di­ethyl­benzo[g]quinoxaline
Source: IUCrdata. 2020 Apr 7;5(Pt 4):x200454. doi: 10.1107/S241431462000454X (PMC9462215; doi:10.1107/S241431462000454X)
Supplement: Supplementary file 4 [file x-05-x200454-sup3.pdf]

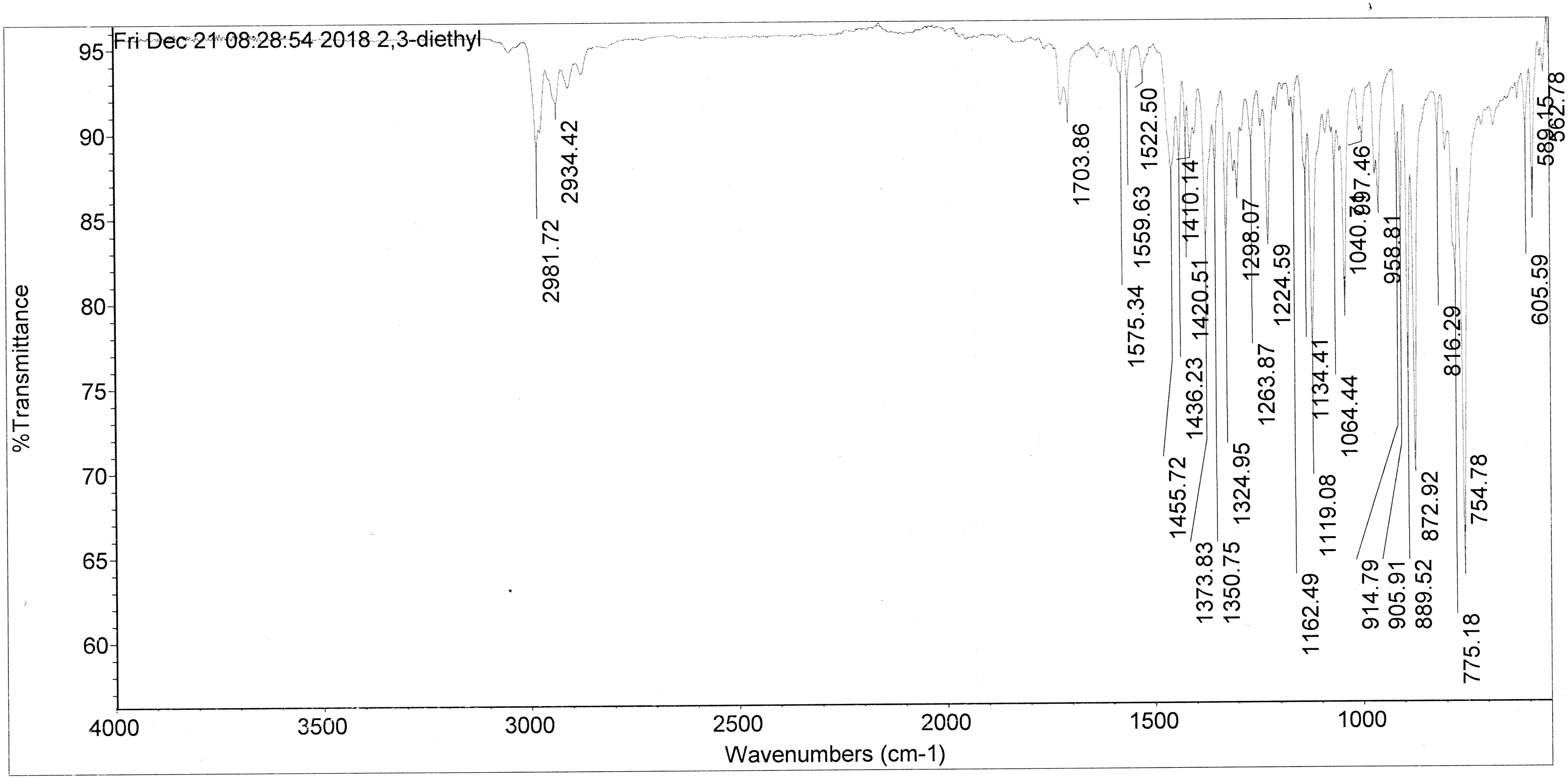

Fri Dec 21 08:31:43 2018 (GMT-05:00)

FIND PEAKS:

Spectrum:	Fri Dec 21 08:28:54 2018 2,3-diethyl
Region:	4000.12 550.10
Absolute threshold:	93.916
Sensitivity:	50
Peak list:	

Position:	562.78	Intensity:	93.539
Position:	589.15	Intensity:	85.868
Position:	605.59	Intensity:	90.178
Position:	754.78	Intensity:	64.763
Position:	775.18	Intensity:	81.815
Position:	816.29	Intensity:	90.112
Position:	872.92	Intensity:	70.004
Position:	889.52	Intensity:	75.691

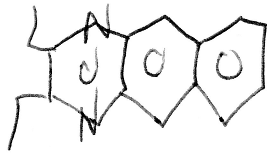

138°c

1.11 g

Position:	905.91	Intensity:	84.925
Position:	914.79	Intensity:	88.247
Position:	958.81	Intensity:	85.696
Position:	997.46	Intensity:	89.699
Position:	1040.71	Intensity:	79.870
Position:	1064.44	Intensity:	87.727
Position:	1119.08	Intensity:	77.599
Position:	1134.41	Intensity:	87.582
Position:	1162.49	Intensity:	90.528
Position:	1224.59	Intensity:	83.377
Position:	1263.87	Intensity:	89.504
Position:	1298.07	Intensity:	86.601
Position:	1324.95	Intensity:	83.569
Position:	1350.75	Intensity:	87.906
Position:	1373.83	Intensity:	83.703
Position:	1410.14	Intensity:	88.514
Position:	1420.51	Intensity:	89.803
Position:	1436.23	Intensity:	88.366
Position:	1455.72	Intensity:	87.714
Position:	1522.50	Intensity:	93.325
Position:	1559.63	Intensity:	92.706
Position:	1575.34	Intensity:	93.175
Position:	1703.86	Intensity:	91.357
Position:	2934.42	Intensity:	91.742
Position:	2981.72	Intensity:	89.214

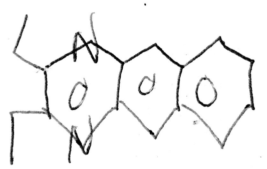

diethylnapthlenequinone

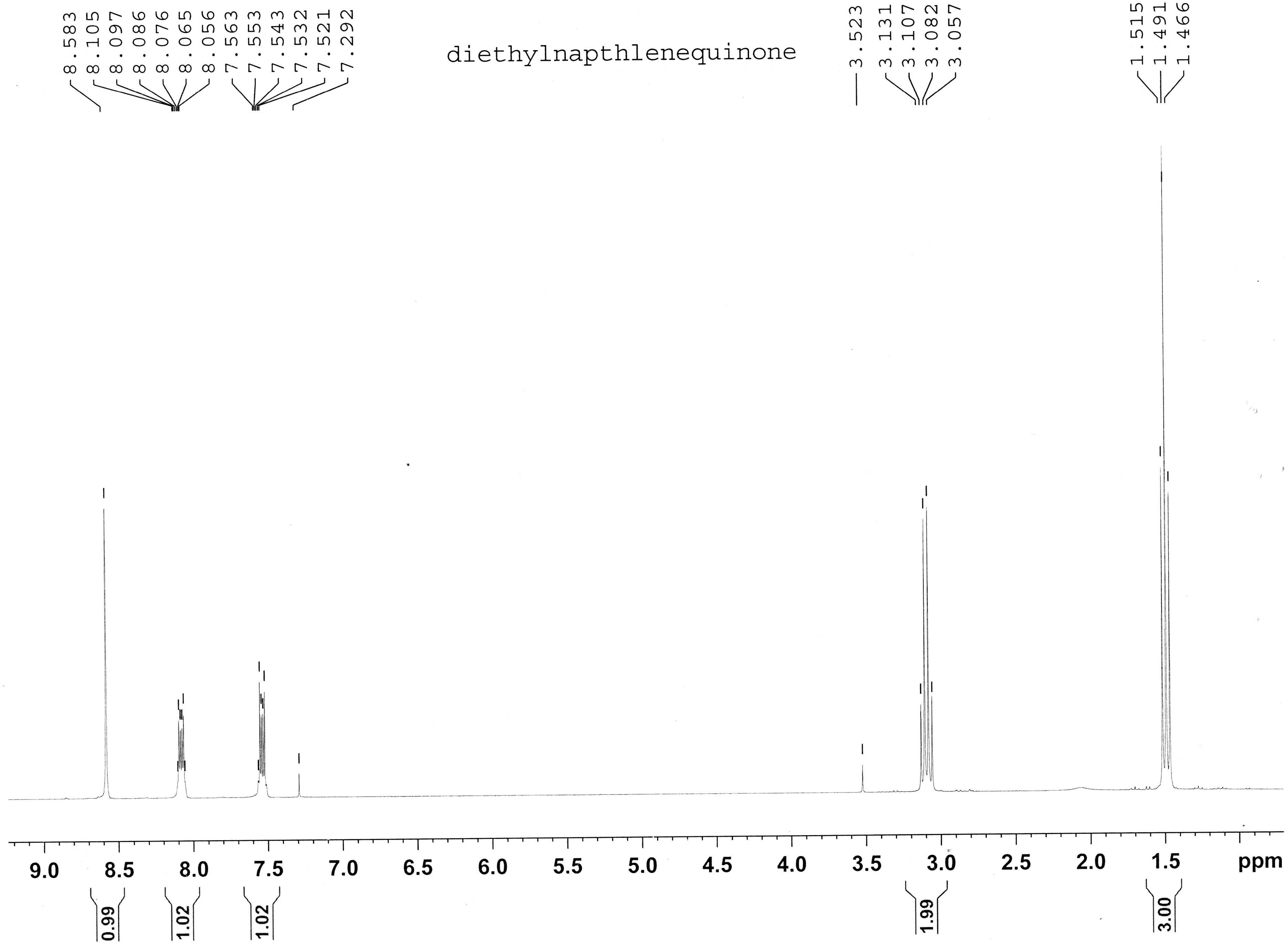

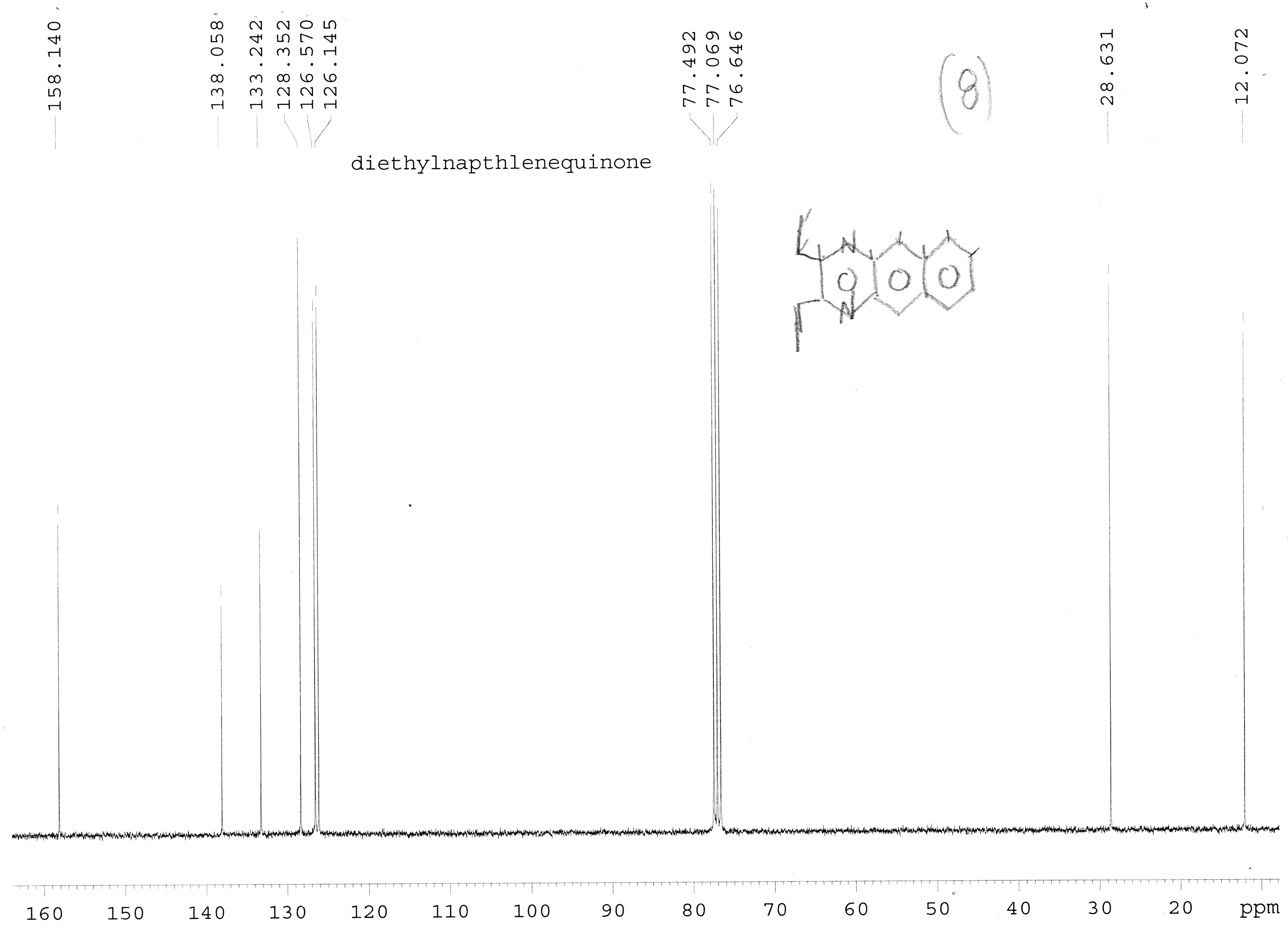
158.140
138.058
133.242
128.352
126.570
126.145
77.492
77.069
76.646
28.631
12.072
diethylnapthlenequinone
(8)
160
150
140
130
120
110
100
90
80
70
60
50
40
30
20
ppm
